# Non coding RNAs as the critical factors in chemo resistance of bladder tumor cells

**DOI:** 10.1186/s13000-020-01054-3

**Published:** 2020-11-12

**Authors:** Amir Sadra Zangouei, Hamid Reza Rahimi, Majid Mojarrad, Meysam Moghbeli

**Affiliations:** 1 Student Research Committee, Faculty of Medicine, Mashhad University of Medical Sciences, Mashhad, Iran; 2 Department of Medical Genetics and Molecular Medicine, School of Medicine, Mashhad University of Medical Sciences, Mashhad, Iran

**Keywords:** Bladder cancer, LncRNA, MiRNA, Drug resistance, Chemotherapy, Prognosis, Marker

## Abstract

**Background:**

Bladder cancer (BCa) is the ninth frequent and 13th leading cause of cancer related deaths in the world which is mainly observed among men. There is a declining mortality rates in developed countries. Although, the majority of BCa patients present *Non-Muscle-Invasive Bladder Cancer* (NMIBC) tumors, only 30% of patients suffer from muscle invasion and distant metastases. Radical cystoprostatectomy, radiation, and chemotherapy have proven to be efficient in metastatic tumors. However, tumor relapse is observed in a noticeable ratio of patients following the chemotherapeutic treatment. Non-coding RNAs (ncRNAs) are important factors during tumor progression and chemo resistance which can be used as diagnostic and prognostic biomarkers of BCa.

**Main body:**

In present review we summarized all of the lncRNAs and miRNAs associated with chemotherapeutic resistance in bladder tumor cells.

**Conclusions:**

This review paves the way of introducing a prognostic panel of ncRNAs for the BCa patients which can be useful to select a proper drug based on the lncRNA profiles of patients to reduce the cytotoxic effects of chemotherapy in such patients.

## Background

Bladder cancer (BCa) is the ninth most frequent cancer worldwide with an annual estimated 356,000 new cases and 145,000 deaths [1]. It ranks the fourth common cancer among males [2]. Many factors are involved in BCa progression such as smoking, industrial carcinogens, and familial history [3]. Approximately 10–20% of the patients who experience recurrence are prone to develop the muscle-invasive bladder cancer (MIBC) [4]. Although, surgery is the main treatment option of non-invasive bladder cancer, a noticeable ratio of these patients experience tumor relapse [3]. Non–muscle-invasive tumors can be treated by transurethral resection followed by chemotherapy or immunotherapy. Grade of tumor invasion is an important factor for the treatment management in which low-grade tumors are treated with only resection, while high-grade with relapse risk may require further resection and bacille Calmette-Guérin (BCG) therapy [5]. Chemotherapy has been considered as an effective first-line treatment for early BCa aiming to suppress cancer progression, prevent recurrences, and enhance patients’ survival [6]. However, BCa is prone for the chemo resistance and tumor relapse. Since, early detection can significantly improve the survival rate, monitoring of the drug-resistance progression can be helpful for early treatment of recurrence [7]. A combine of chemo radiation and cystectomy, offers an efficient option with long-term survival rates [8]. The methotrexate, vinblastine, doxorubicin, and cisplatin combination therapy was associated with severe side effects, while the Gemcitabine/cisplatin combination is safe and efficient in BCa patients [9]. Cisplatin-based chemotherapy is the common method, however it has not any influence on overall survival following radical cystectomy among high-risk cases [10]. Regarding the chemotherapeutic resistance, many patients are faced with side effects without any efficient benefit. Genetic factors are associated with drug resistance through regulation of drug efflux, DNA repair, cell cycle, and apoptosis [11–13]. Non-coding RNAs (ncRNAs) are a class of RNAs including long non-coding RNAs (lncRNA), micro RNAs (miRNA), and circular RNAs (circRNA) which are involved in post transcriptional regulation. Since, the ncRNAs have an important role in drug response of tumor cells [14–16], we have summarized all of the reported ncRNAs which have been associated with chemotherapeutic resistance in BCa for the first time in the world (Table.1).

**Table 1:** all of the ncRNAs associated with chemotherapeutic resistance in BCa

Study	Year	Gene	Country	Drug	Results
Chen [17]	2020	MST1P2, miR-133b	China	Cisplatin	MST1P2/miR-133b axis had an important role in cisplatin-resistance of bladder cancer through SIRT1/p53 pathway.
Chen [18]	2019	HIF1A-AS2	China	Cisplatin	HIF1A-AS2 enhances survival of tumor cells by upregulating HMGA1.
Wu [19]	2019	UCA1	China	Rapamycin	UCA1 acts as an endogenous sponge to down regulate the miR-582-5p which resulted in ATG7 over expression.
Zhang [20]	2017	GAS5	China	Doxorubicin	GAS5 increased doxorubicin-induced apoptosis through BCL-2 suppression.
Liu [21]	2019	MALAT1	China	Cisplatin	MALAT1 induced chemo resistance through regulating miR-101-3p/VEGFC axis.
Li [22]	2019	GHET1	China	Gemcitabine	GHET1 up regulated the MRP1.
An [23]	2018	FOXD2-AS1	China	Gemcitabine	FOXD2-AS1 indirectly targets the ABCC3 through miR-143 sponging.
Wang [24]	2017	MiR-143	China	Gemcitabine	The miR-143 attenuated gemcitabine resistance via IGF-1R suppression.
Fan [25]	2014	UCA1	China	Cisplatin	UCA1 overexpression was contributed to upregulation of WNT6.
Pan [26]	2016	UCA1	China	Cisplatin, Gemcitabine	UCA1 activates miR-196a-5p via CREB which results in gemcitabine/cisplatin resistance.
Xie [27]	2017	TUG1	China	Doxorubicin	TUG1 knockdown decreased Dox resistance through restraining the activity of Wnt/β-catenin pathway.
Xie [28]	2018	CDKN2B-AS	China	Gemcitabine	CDKN2B-AS induced Gemcitabine-resistance via sponging Let-7.
Zhuang [29]	2017	LET	China	Gemcitabine	TGFβ1 promotes gemcitabine resistance through LncRNA-LET/NF90/miR-145 axis.
Li [30]	2019	DLEU1	China	Cisplatin	DLEU1 up regulated the HS3ST3B1 via miR-99b suppression.
Zhao [31]	2019	NEAT1.1	China	Cisplatin	NEAT1.1 was down regulated following cisplatin treatment.
Xiao [32]	2018	MiR-22-3p	China	Paclitaxel, Adriamycin, Epirubicin, hydroxycamptothecin, Cisplatin, and Gemcitabine	MiR-22-3p enhanced resistance to chemotherapy in bladder tumor cells through suppressing NET1.
Deng [33]	2015	MiR-27a	China	Paclitaxel, Adriamycin, Cisplatin	MiR-27a/RUNX-1 pathway has a key function in chemo-resistance.
Drayton [34]	2014	MiR-27a	UK	Cisplatin	MiR-27a deregulation induced cisplatin resistance in bladder cancer cells via up regulating SLC7A11.
Bu [35]	2014	MiR-101	China	Cisplatin	MiR-101 regulates cisplatin sensitivity in bladder tumor cell lines via targeting the COX-2.
Vinall [36]	2012	MiR-34a	USA	Cisplatin	MiR-34a sensitized tumor cells to cisplatin by targeting SIRT-1 and CDK6.
Li [37]	2014	MiR-34a	China	Cisplatin	MiR-34a targets CD44 after cisplatin therapy.
Liu [38]	2018	MiR-34a	China	Epirubicin	MiR-34a significantly reduced Epirubicin chemo resistance in bladder tumor cells through targeting TCF1 and LEF1.
Zhang [39]	2017	MiR-34a	China	Cisplatin, Gemcitabine	MiR34a regulation of GOLPH3 is active in bladder CSCs resistant to gemcitabine and cisplatin.
Tan [40]	2019	MiR-34b-3p	China	Paclitaxel, Adriamycin, Epirubicin, Cisplatin, Pirarubicin	MiR-34b-3p attenuated chemo resistance in bladder cancer through suppressing CCND2 and P2RY1.
Luan [41]	2018	MiR-98	China	Cisplatin, Doxorubicin	MiR-98 promotes chemo-resistance through targeting LASS2.
Li [42]	2019	MiR-101-3p	China	Cisplatin	MiR-101-3p decreased cisplatin-resistance in bladder urothelial carcinoma through repressing EZH2 and MRP1.
Cao [43]	2018	MiR-129-5p	China	Gemcitabine	MiR-129-5p inhibits resistance to gemcitabine in bladder cancer cells and promotes their apoptosis via targeting WNT5a.
Lv [44]	2015	MiR-193a-3p	China	Pirarubicin, Paclitaxel, Adriamycin, Epirubicin Hydrochloride, and Cisplatin	MiR-193a-3p mediated HOXC9 down regulation which resulted in poorer sensitivity to chemotherapeutic drugs.
Deng [45]	2015	MiR-193a-3p	China	Pirarubicin, Paclitaxel, Adriamycin, Epirubicin hydrochloride, and Cisplatin	PSEN1 was directly targeted by miR-193a-3p and executed its impact on the multi-chemo resistance.
Lin [46]	2017	MiR-193b-3p	Taiwan	Cisplatin	CEBPD/miR-193b-3p axis had key roles in cisplatin response.
Deng [47]	2014	MiR-193a-3p	China	Paclitaxel, Adriamycin, Epirubicin Hydrochloride, and Cisplatin	MiR-193a-3p induced multi-drug resistance in bladder cancer cells through down regulating LOXL4.
Lv [48]	2014	MiR-193a-3p	China	Pirarubicin hydrochloride, Paclitaxel, Adriamycin, and Epirubicin hydrochloride	HIC2, SRSF2, and PLAU achieve their role in relaying miR-193a-3p’s effect on chemo resistance in bladder cancer through regulation of Myc/Max, NF-jB, DNA damage response, and NOTCH pathway.
Shindo [49]	2018	MiR-200b	Japan	Cisplatin	TNFSF10, ICAM1, and IGFBP3 were induced in the resistant cells as a result of miR-200b + cisplatin treatment.
Zhang [50]	2015	MiR-203	China	Cisplatin	The miR-203 up regulation increased the cytotoxic effects of cisplatin and decreased tumor cell viability through suppressing Survivin and BCL-w.
Liu [51]	2018	MiR-214	China	Cisplatin	MiR-214 decreased chemo resistance in bladder cancer tissues and cell lines by suppressing NTN1.
Li [52]	2017	MiR-218	China	Cisplatin	MiR-218 up regulation reduced cisplatin resistance through GLUT1 targeting.
Zeng [53]	2016	MiR-222	China	Cisplatin	MiR-222 up regulation decreased cisplatin-induced apoptosis in bladder tumor cells through modulation of PPP2R2A/Akt/mTOR pathway.

## Main text

### Long non coding RNAs

LncRNAs are key regulatory molecules involved in cell proliferation, development, and oncogenesis that achieve their roles through post-transcriptional regulation [54]. They have pivotal roles in transcriptional regulation through functioning as molecular signals, sponges, decoys, scaffolds, and enhancer RNAs [55, 56]. Drug resistance in various malignancies can be attributed to lncRNAs function as regulator of gene expression which results in higher rate of tumor cell proliferation and reduced apoptosis [57]. Cisplatin (DDP) has been used among the first-line chemotherapy medications for high grade and stage bladder tumor patients [58]. However, a large fraction of BCa patients are resistant to cisplatin-based chemotherapy [9, 59]. Sirtuin-1 (SIRT1) is a NAD-dependent deacetylase that diminishes the tumor suppressive effect of p53, thereby dampening the efficacy of clinical radiotherapy and chemotherapy. Therefore, SIRT1 inhibition results in tumor cells death through p53 modulation and activation [60]. It has been reported that there were significant decreased and increased levels of *miR-133b* and *MST1P2* expressions in cisplatin-resistant bladder tumor cell lines, respectively. *MiR-133b* directly suppressed the *SIRT1* expression. MST1P2/miR-133b axis had an important role in cisplatin-resistance of BCa through SIRT1/p53 pathway [17]. It has been shown that there was overexpression of *HIF1A-AS2* in tissues and cell lines of BCa following the cisplatin treatment which makes bladder tumor cells resistance to cisplatin-triggered cell death. *HIF1A-AS2* enhanced survival of tumor cells by upregulating high-mobility group A1 (HMGA1), thereby limiting the transcriptional function of p53 family. It was identified that interaction of p53 with HMGA1 restricted their transcriptional activity on proapoptotic BAX protein [18]. *MiR-582-5p* has tumor-suppressive functions and reduces the tumor cell proliferation and migration via targeting *CDK1*, *FOXC1*, and *RAB27a* [61–63]. ATG7 is implicated in the two ubiquitin-like systems and is essential for autophagy [64]. It has been reported that the *UCA1* was up regulated in BCa. It acts as an endogenous sponge to down regulate the *miR-582-5p* which resulted in *ATG7* over expression. *UCA1* is important for the regulation of proliferation and invasion of BCa cells through modulating UCA1-miR-582-5p-ATG7-autophagy axis. As *UCA1* shRNA markedly reduced the expression level of LRP, MRP1, and GST, and significantly overexpressed TOPO-II, it is hypothesized that knockdown of *UCA1* decreases chemo resistance [19]. It has been observed that there was *GAS5* down regulation in bladder transitional cell carcinoma which was associated with advance grade and stage. *GAS5* also increased doxorubicin-induced apoptosis through *BCL-2* suppression [20].

Drug efflux is also another mechanism of tumor drug resistance that can be regulated by different lncRNAs [65]. *MALAT1* increases the expression levels of *MRP1* an *MDR1* through STAT3, thereby is responsible for inducing cisplatin-resistance in lung tumor cells [66]. It has been shown that *MALAT1* repression caused a better response of BCa cells to chemotherapy and increased cisplatin sensitivity. *MALAT1* induced chemo resistance through regulating miR-101-3p/VEGFC axis. Bladder tumor tissue had higher level of *MALAT1* compared with normal margins [21]. Gastric carcinoma proliferation-enhancing transcript 1 (GHET1) is a lncRNA involved in cisplatin resistance in gastric cancer [67]. MRP1 is a member of ATP-binding cassette (ABC) superfamily which regulates the intracellular distribution of molecules and is also involved in transport of different complexes across extra-and intra-cellular membranes. Moreover, it confers resistance to chemotherapeutic treatments in cancer cells due to its ability of drugs efflux. It has been observed that there was significant *GHET1* overexpression in BCa, which was positively correlated with advance tumor grade and muscle invasion. *GHET1* up regulation was also associated with higher Gemcitabine-chemo resistance in BCa cells. Moreover, *GHET1* up regulated the MRP1 in BCa cells, which in turn enhanced their Gemcitabine resistance [22]. Gemcitabine is a nucleotide analogue commonly used as the first line anticancer drug therapy for many solid tumors such as breast cancer, ovarian cancer, and BCa [68, 69]. *FOXD2-AS1* is significantly up regulated in BCa, and via establishing a positive feedback loop with AKT and E2F1 is contributed to increased progression and aggressiveness of bladder tumor cells [70]. It has been shown that there was a dose-dependent pattern of *FOXD2-AS1* overexpression in gemcitabine resistant bladder tumor cells. Repression of *FOXD2-AS1* expression resulted in lower levels of ABCC3 protein, and down regulation of several genes related to inducing drug resistance including *MDR1*, *MRP2*, and *LRP*. Therefore, *FOXD2-AS1* regulated gemcitabine-resistance in BCa cells. *FOXD2-AS1* indirectly targets the ABCC3 through *miR-143* sponging [23]. Insulin-like growth factor-1 receptor (IGF-1R) has pivotal role in cell survival, differentiation, proliferation, and apoptosis inhibition [71]. IGF-1R activates the PI3K/AKT signaling which is critical for cell survival [72, 73]. *MiR-143* up regulation suppresses tumor cells proliferation and migration, and triggers apoptosis. It also increases the oxaliplatin sensitivity of tumor cells through targeting IGF-1R [74]. It has been reported that there was significant decreased levels of *miR-143* expression in bladder tumor cell lines and tissue samples compared with normal margins. There was an inverse association between *miR-143* and *IGF-1R* mRNA expression levels which showed that the *miR-143* exerts its tumor-suppressive role through IGF-1R regulation. *MiR-143* overexpression significantly inhibited the p-ERK and p-AKT levels. It also attenuated the gemcitabine resistance via IGF-1R suppression [24].

LnRNAs can also be associated with drug response during tumor progression through regulation of different signaling pathways [75]. Studies have confirmed the association between up regulation of lncRNA urothelial carcinoma associated 1 (UCA1) in bladder tumor tissue with cell growth, invasion, and migration [76]. *UCA1* is frequently up regulated in bladder malignancies and contributed to aggressiveness of bladder tumor cells [76]. WNT signaling is an important pathway during embryogenesis and carcinogenesis [77, 78]. A significant *UCA1* up regulation has been shown in bladder tumor tissues following cisplatin treatment. *UCA1* overexpression was also contributed to up regulation of WNT6 and induction of WNT pathway which promotes cisplatin resistance in tumor cells [25]. The WNT6 and SRPK1 are up regulated as a result of *UCA1* overexpression, which leads to cisplatin resistance [25, 79]. *UCA1* promotes epithelial-mesenchymal transition (EMT) and activates mTOR and ERK pathways, and increases Gefitinib resistance in EGFR-mutant lung carcinoma [80]. It has been reported that the *UCA1* activates *miR-196a-5p* via CREB which results in gemcitabine/cisplatin resistance. *UCA1* up regulation had a significant association with diminished rate of apoptosis and higher cell survival. *UCA1* promotes CREB phosphorylation through AKT pathways. Therefore, UCA1-dependent CREB activation was considered as a key step in *miR-196a-5p* transcriptional regulation in bladder tumor cells [26]. *TUG1* is an oncogenic lncRNA in various cancers [81–86]. Doxorubicin (Dox) is an anthracycline antibiotic which induces cell cycle arrest and apoptosis through induction of the double-strand breaks [87]. It has been reported that the *TUG1* knockdown decreased Dox resistance through restraining the activity of Wnt/β-catenin pathway; whereas, *TUG1* up regulation was significantly associated with Dox resistance and poor prognosis [27]. *CDKN2B-AS* is an oncogenic lncRNA in various cancers [88–90]. Gemcitabine is a deoxycytidine analogue with anticancer function, which is used as the first-line chemotherapeutic medication against bladder urothelial carcinoma. It is metabolized and activated by cytidine deaminase and deoxycytosine kinase, respectively. It disrupts the replication of DNA, causes cell cycle arrest at G1/S stage, and promotes apoptosis [91]. It has been reported that there was up regulation of *CDKN2B-AS* in bladder urothelial carcinoma tissues and cell line which was positively associated with advance tumor grade. *CDKN2B-AS* up regulation was also correlated with Gemcitabine chemo resistance in BCa patients. Suppression of *CDKN2B-AS* attenuated the Gemcitabine-resistance in 24/Gem cells through inactivation of WNT signaling pathway. Therefore, *CDKN2B-AS* induced Gemcitabine-resistance via sponging Let-7 for activating WNT signaling pathway [28]. Although, anti-neoplastic chemotherapeutic drugs like gemcitabine at first show beneficial effects in almost all patients, a noticeable ratio of patients experience recurrences following resistance. TGFβ1 is a cytokine involved in EMT and self-renewal [92]. NF90 is also a RNA binding protein with critical roles in RNA processing, localization, turnover, and transcriptional stability of *HIF-1α*, *IL-2*, and *VEGF* [93–96]. The up regulation of CSC markers such as CK14, CK5, and CD44 indicated that the BCa stemness is stimulated during chemotherapy. *TGFβ1* was overexpressed following Gemcitabine treatment. Moreover, aberrant expression of lncRNA-LET/NF90/miR-145 pathway was mediated by TGFβ1 which eventually increased stemness and chemo resistance. KLF4 and HMGA2 as the *miR-145* targets were responsible for *miR-145* suppressive effect against the stemness of BCa cell [29].

The *DLEU1* is an lncRNA associated with tumor cell aggressiveness and migration [97–100]. It has been shown that there were higher levels of *DLEU1* expressions in bladder tumor tissues compared with normal margins. *DLEU1* up regulation was also associated with worse prognosis in BCa patients. Moreover, up regulation of *DLEU1* enhanced tumor growth and aggressiveness and induced cisplatin resistance through HS3ST3B1 induction. *DLEU1* up regulated the *HS3ST3B1* via *miR-99b* suppression. Overexpression of HS3ST3B1 was significantly correlated with shorter survival rates in BCa patients. Furthermore, ectopic HS3ST3B1 expression enhanced tumor growth, invasiveness, and cisplatin resistance [30]. *NEAT1* is an oncogenic lncRNA in various cancers and promotes the cell proliferation, migration, and aggressiveness through regulation of various miRNAs. Some studies have demonstrated that *NEAT1* attenuates cisplatin chemo resistance [101, 102], while other studies showed the *NEAT1* as inducer of cisplatin resistance [103, 104]. It has been demonstrated that the *NEAT1.1* was down regulated following cisplatin treatment in BCa cells. The p53, OCT4, and c-MYC regulated the expression level of *NEAT1* through interacting with its promoter region. There were OCT4, c-MYC, and p53 up regulations in cisplatin-resistant BCa cells. The knockdown of *NEAT1* suppressed the proliferation and migration of BCa cells and induced apoptosis following cisplatin treatment [31].

### MicroRNA-22-3p and 27a

Neuroepithelial cell transforming 1 (NET1) is a guanine nucleotide exchange factor for RhoA and is involved in regulating extracellular signal transduction. It has been observed that the *miR-22-3p* enhanced resistance to chemotherapy in bladder tumor cells through suppressing NET1. NET1 was also markedly up regulated in 5637 cell line compared with H-bc cell line. Moreover, there was an inverse association between *NET1* and *miR-22-3p* expression levels. NET1 was introduced as the direct target of *miR-22-3p* in chemo resistance of bladder tumor cells [32]. *MiR-27a* down regulation in bladder tumors can be associated with reduced chemotherapeutic response [34, 105–107].

RUNX-1 is a direct target for inducing tumor chemo-sensitivity using *miR-27a*. The findings have shown a significant correlation between *miR-27a* overexpression and improved chemotherapeutic outcomes. The carriers of rs11671784 A allele had significantly poorer outcomes after chemotherapy compared with rs11671784 GG homozygote patients. It was also indicated that the *miR-27a* significantly down regulated the P-glycoprotein [108]. It has been reported that the *miR-27a* up regulation was significantly associated with overexpression of CASP3 and BAX, and BCL-2 down regulation. *MiR-27a* decreased tumor cells’ resistance to chemotherapy by increased rate of apoptosis. There was a correlation between rs11671784 G/A variation and reduced *miR-27a* expression which results in increased RUNX-1 expression drug resistance. RUNX-1 up regulation was significantly correlated with reduced bladder tumor drug sensitivity. Therefore, miR-27a/RUNX-1 pathway has a key function in chemo-resistance in bladder malignancies [33]. Many solid tumors display resistance towards cisplatin mainly due to sequestration, reduced uptake, and increased drug efflux. Sequestration of cisplatin is accomplished by a variety of substances such as glutathione (GSH) as an efficient electron donor involved in detoxification of xenobiotics [109]. Glutathione shows antagonistic effect against cytotoxicity of radiotherapy and chemo therapeutic medication [110]. Glutamate-cysteine ligase catalyzes the first step of GSH synthesis that is regulated by the availability of cystine at both transcription and translation levels [111]. Heterodimeric xc- cystine-glutamate transporter is an antiporter which simultaneously exports glutamate and imports cystine [112], and is consisted of SLC3A2 and SLC7A11. It has been observed that the *miR-27a* deregulation induced cisplatin resistance in BCa cells via up regulating SLC7A11, followed by increased cystine import and higher intracellular glutathione levels. The results suggested that the *miR-27a/27b* and *SLC7A11* expression levels along with intracellular glutathione levels in BCa tissue could be considered as predictive factor for determining the probability of cisplatin-chemo resistance. Patients with down regulated SLC7A11 showed better response to therapy and had better prognosis [34]. Cyclooxygenase-2 (COX-2) is an important mediator for the synthesis of inflammatory prostaglandins and is involved in tumor invasion, angiogenesis, and drug resistance [113, 114]. There is a negative correlation between *miR-101* and *COX-2* expressions in which *miR-101* up regulation reduces cisplatin-chemo resistance through *COX-2* inhibition [115]. It has been shown that there was a significant decreased expression of *miR-101* in BCa cells resistant to cisplatin. Therefore, *miR-101* regulates cisplatin sensitivity in bladder tumor cell lines via targeting the *COX-2* [35].

### MicroRNA-34a and 98

*MiR-34a* is a potential tumor suppressor miRNA and its down regulation has been reported in various malignancies [116]. Dysregulated *miR-34a* has been associated with resistance to chemotherapeutic drugs [36, 117–120]. This might be due to the modulating impact of *miR-34a* on p53 signaling pathway. Ectopic expression of *miR-34a* caused apoptosis, cell cycle arrest, and drug response alteration through *SIRT-1*, *CDK6*, *E2F3*, and *BCL-2* targeting [121–123]. CD44 is considered as the marker of chemo-resistant bladder CSCs [124–126]. It has been reported that there was a correlation between *miR-34a* up regulation and cisplatin sensitivity in BCa. Moreover, *miR-34a* targets *CD44* after cisplatin therapy [37]. It has been reported that up regulation of *miR-34a* significantly reduced Epirubicin chemo resistance in bladder tumor cells through targeting *TCF1* and *LEF1*. Therefore, *miR-34a* up regulation leads to the suppression of WNT signaling pathway while increasing the rate of epirubicin -induced apoptosis [38]. Golgi phosphoprotein 3 (GOLPH3) is involved in Golgi trafficking [127]. GOLPH3 deregulation is associated with poor prognosis in BCa [128]. It has been reported that there was a significant reduced *miR-34a* expression in gemcitabine-resistant BCa cells. *MiR-34a* reduced the stemness of chemo resistant BCa cells and increased gemcitabine and cisplatin responses. GOLPH3 was also significantly over expressed in BCa cells, xenograft, and sphere cells resistant to gemcitabine and cisplatin. Moreover, the up regulation of CSC biomarkers including *KLF4*, *SOX2*, and *CD44* were observed in bladder tumor cells and xenograft. Therefore, *miR-34a* regulation of GOLPH3 is active in bladder CSCs resistant to gemcitabine and cisplatin [39]. Aberrant p53/Rb signaling pathway is correlated with increased tumor invasiveness and growth in muscle invasive BCa [129–131]. The expression levels of components of this pathway are important in predicting the clinical outcome of the chemotherapy. E2Fs as downstream effectors of Rb, and CDK6 as regulator of Rb phosphorylation are directly targeted by *miR-34a*. *MiR-34a* enhances apoptosis rate through suppressing *BCL-2* expression. CDK6 interacts with CDK4 and CCND1 to form a complex which is fundamental for Rb function and G1/S transition. SIRT-1 is a NAD-dependent deacetylase which targets FOXO, SFRP1, p53, and PGC1 [132–134]. It has been reported that the *miR-34a* sensitized tumor cells to cisplatin by targeting SIRT-1 and CDK6. The MI-TCC patients resistant toward cisplatin chemotherapy had significantly lower levels of *miR-34a* expression compared with sensitive patients [36]. Cyclin is critical for the regulation of cyclin-dependent kinase (CDK) activity. Cyclin D-CDK4/6 complex has a critical role during transition from G1 to S phase [135, 136]. The P2RY1 is a member of G-protein-coupled receptors family which is a receptor for extracellular ADP [137, 138]. Binding of ADP to P2RY1 mobilizes intracellular calcium through activation of phospholipase C, which results in platelet shape change and aggregation [139, 140]. It has been reported that the *miR-34b-3p* attenuated chemo resistance in BCa through suppressing *CCND2* and *P2RY1* [40].

*MiR-98* was recognized as an important agent in regulating mitochondrial activity, which increases bladder tumor cells resistance toward mitochondrial apoptosis. It was also established that *miR-98* targets *LASS2* tumor suppressor. There was also an inverse association between *miR-98* and *LASS2* mRNA levels in bladder tumors. LASS2 functions in negative regulation of mitochondrial activity and has a putative role in mediating chemo-resistance caused by *miR-98*. Therefore, *miR-98* promotes chemo-resistance through targeting *LASS2*, which enhances mitochondrial fusion and disrupts mitochondrial membrane potential [41].

### MicroRNA-101, 129-5p, and 193a-3p

*MiR-101-3p* is considered as a tumor suppressor and is down regulated in different malignancies such as BCa, colorectal cancer, and breast cancer [141–144]. EZH2 as a target of *miR-101-3p* is a member of the Polycomb-group family involved in transcriptional repression [145]. MRP1 is a member of ATP-binding cassette (ABC) transporters which transport different molecules across intra- and extra-cellular membranes. It induces chemo resistance via exporting chemotherapeutic medications before they exert their antineoplastic effects [146–148]. It has been reported that there were *miR-101-3p* down regulations in bladder urothelial carcinoma tissues and cell lines resistant to cisplatin. *MiR-101-3p* overexpression also suppressed the MRP1 expression level. Therefore, *miR-101-3p* decreased cisplatin-resistance in bladder urothelial carcinoma through repressing EZH2 and MRP1 [42]. Gemcitabine is a deoxycytidine analogue which disrupts DNA synthesis, induces replication-associated DNA double-strand breaks, and triggers apoptosis in cancer cells. Gemcitabine is effective in improving overall survival (OS) in metastatic BCa patients [149].

NOTCH signaling pathway has a pivotal role in various cellular processes such as cell cycle, migration, metabolism, and apoptosis [150, 151]. *MiR-129-5p* is involved in tumor cells drug response via modulation of NOTCH signaling receptor DLK1 [152]. WNT5a is also a member of WNT ligand family, which has critical role is regulation of cell proliferation and migration [24]. WNT5a increases the GSK-3-independent degradation of β-catenin [153–155]. Some studies have revealed that WNT5a induces resistance to chemotherapy via up regulating ABCB1 [156] and inducing PI3K/AKT signaling pathway [157]. It has been reported that lower levels of *miR-129-5p* was correlated with lower sensitivity of BCa cells to gemcitabine therapy; however, overexpression of *miR-129-5p* inhibits resistance to gemcitabine in BCa cells and promotes their apoptosis via targeting WNT5a [43].

Substantial epigenetic changes along with genetic variations are the origin of all cancerous features [158]. These epigenetic changes and defects have a more significant impact on tumor cells phenotype and gene expression than genetic changes. Detection of aberrant DNA methylation at promoter sequence of oncogene and tumor suppressor genes is an efficient method of early diagnosis [159–161]. *MiR-193a-3p* impedes tumor proliferation and decreases drug resistance through down regulation of various genes such as *CCND1*, *ERBB4*, and *PTEN* [162, 163]. *HOXC9* belongs to highly conserved homeobox family of genes, and encodes proteins that function as homeodomain transcription factors playing a crucial role in morphogenesis in all multicellular organisms. It has been revealed that the *miR-193a-3p* mediated *HOXC9* down regulation which resulted in poorer sensitivity of BCa to chemotherapeutic drugs. Oxidative stress and DNA damage response were also influenced by epigenetic suppression of *HOXC9* through *miR-193a-3p* [44]. Presenilin (PSEN1) is a catalytic element of the γ-secretase complex that performs intramembrane cleavage of numerous protein substrates leading to activation of the NOTCH pathway [164]. Studies have shown the positive impact of PSEN1 on overexpression of ABCC1/MRP1 via NOTCH signaling [165]. It has been reported that the *PSEN1* was directly targeted by *miR-193a-3p* and executed its impact on the multi-chemo resistance. PSEN1 up regulation rendered H-bc cells more sensitive to chemotherapy-induced cell death. However, RNA interference-mediated repression of PSEN1 gene resulted in lower rates of apoptosis and desensitizing 5637 cell line to chemotherapy-induced cell death [45]. Platinum compounds are frequently used for treatment of various malignancies by forming bifunctional DNA adducts which results in transcriptional suppression and apoptosis induction [46]. C/EBP is a family of transcription factors with pivotal roles in regulation of cellular differentiation, proliferation, and apoptosis [166–168]. CEBPD is also involved in genomic stability through transcriptional modulation of DNA damage response proteins. It was observed that CEBPD and cisplatin increased the expression levels of *miR-193b-3p*. Moreover, *miR-193b-3p* had regulatory effect on ETS1 and CCND1. *MiR-193b-3p* was also important for CDDP-triggered cell cycle arrest, cell cytotoxicity, and inhibition of cellular migration. CEBPD/miR-193b-3p axis had key roles in cisplatin response of urothelial carcinoma cells in which CEBPD up regulates the *miR-193b-3p* and improved cisplatin cytotoxicity in urothelial carcinoma. This process was associated with ETS1 and CCND1 down regulations, cell migration inhibition, cell cycle arrest, and cisplatin-triggered cytotoxicity in NTUB1 cell line [46]. The oncogenic function of *miR-193a-3p* is due to its suppressive effects on various genes such as KRAS and c-KIT [169, 170]. Lysyl oxidase homolog 4 (LOXL4) is a member of the lysyl oxidase family which is necessary for the biogenesis of connective tissue by formation of crosslinks between collagens and elastin fibers. It has been indicated that the *miR-193a-3p* induced multi-drug resistance in BCa cells through down regulating LOXL4, and thus initiating oxidative stress pathway [47]. Hypermethylation of the promoter and enhancer regions are associated with epigenetically silenced status of ncRNAs and protein-coding genes. The PLAU encodes the urokinase-type plasminogen-activator protein, a serine protease which has key functions in degradation of extracellular matrix during tumor progression and metastasis. The HIC2 is a transcription factor involved in systemic lupus erythematosus [171] and digeorge syndrome [172]. SRSF2 belongs to the serine/arginine-rich family of pre-mRNA splicing factors [173]. HIC2 interacts with CCNT1 to positively regulate MYC/Max pathway [174, 175]. It has been reported that the HIC2, SRSF2, and PLAU achieve their role in relaying *miR-193a-3p*’s effect on chemo resistance in BCa through regulation of Myc/Max, NF-jB, DNA damage response, and NOTCH pathway [48].

### MicroRNA-200b, 203, 214, 218, and 222

Members of *miR-200* family are potent inhibitors of EMT through inhibiting the expression of ZEB1 and ZEB2 [176, 177]. IGFBP3 is an important mediator of insulin growth factor (IGF) signaling pathway. IGF1 shows higher affinity for interaction with IGFBP3 than its specific receptor (IGF1R), thereby IGF1’s binding to IGFBP3 interrupts accurate interaction between IGF1 and IGF1R, dampening the anti-apoptotic functions of IGF1 [178]. TNFSF10 belongs to the TNF superfamily and promotes the apoptosis of tumor cells through activation of death receptors [179]. It has been reported that there was a correlation between epigenetic silencing of *miR-200b* and cisplatin resistance in BCa. Microarray analysis showed that genes associated with CDDP sensitivity or cytotoxicity, such as TNFSF10, ICAM1, and IGFBP3 were induced in the resistant cells as a result of miR-200b/cisplatin treatment [49].

Although, Cisplatin is the main drug in BCa combination chemotherapy regimens including GC (gemcitabine and cisplatin) and MVAC (methotrexate, vinblastine, doxorubicin, and cisplatin), almost half of the MIBC patients do not respond to the cisplatin-based treatment [180]. BCL-w exerts its anti-apoptotic effects through regulation of the intrinsic apoptotic pathway [181]. Anti-apoptotic activity of survivin is accomplished through blocking the caspases in a complex with XIAP [182]. It has been reported that there was a correlation between *miR-203* down regulation and poor prognosis in BCa patients who were under cisplatin-based chemotherapy. *MiR-203* up regulation also increased the cytotoxic effects of cisplatin and decreased tumor cell viability through suppressing Survivin and BCL-w. Patients with non-progressive form of BCa had notably higher levels of *miR-203* compared with cases with progressive form [50].

*MiR-214* functions as a tumor suppressor agent and is deregulated in various cancers [183–186]. Down regulation of *miR-214* occurs in BCa which shows marked correlation with higher tumor grade/stage and lymph nodes involvement [187]. *MiR-214* down regulates the P53 and PDRG1 in BCa [187]. It has been revealed that there was a significant decreased level of *miR-214* expression in BCa tissues and cell lines. The *miR-214* attenuated chemo resistance through apoptosis induction. It was able to down regulate the PARP and CASP-3 levels. It also inhibited AKT phosphorylation. AKT signaling pathways regulates chemotherapy-induced apoptosis via BCL-2 up regulation. Therefore, the impact of *miR-214* on drug resistance is mediated through its modulatory function in AKT/BCL-2 axis. The *miR-214* decreased chemo resistance in BCa tissues and cell lines by suppressing NTN1 [51].

Cisplatin mainly functions through induction of oxidative stress [188]. Nevertheless, a large fraction of tumors develop cisplatin chemo resistance through reducing drug uptake, increasing drug efflux, inactivating ROS, and increasing the intracellular level of GSH [189]. GLUT1 is a uniporter facilitating the transport of glucose across the plasma membrane and mediates glycolytic flux in cells [190]. It has been reported that the *miR-218* up regulation markedly decreased glucose uptake through GLUT1 targeting. Over expression of *miR-218* was also beneficial in attenuating cisplatin resistance in BCa cells [52].

Aberrant expression of *miR-222* enhances tumor cell proliferation and metastasis by inhibiting the PPP2R2A, TIMP3, and p27 [191–193]. *MiR-222* expression has been linked to the tumor drug response [194]. PP2A is regarded as a master regulator of cell cycle and is also involved in the regulation of protein synthesis, apoptosis, and stress responses [191, 195–197]. It has been reported that the *miR-222* up regulation increased cell proliferation and decreased cisplatin-induced apoptosis in bladder tumor cells through modulation of PPP2R2A/AKT/mTOR pathway. Tumor cells with high levels of *miR-222* had activated AKT/mTOR axis. The mTOR or AKT suppression were also beneficial in inhibiting tumor cells’ proliferation and restoring cisplatin sensitivity due to miR-222 up regulation [53].

## Conclusions

Regarding the importance of ncRNAs in regulation of drug response in tumor cells, in present review we have summarized all of the reported ncRNAs which are associated with chemotherapeutic resistance in BCa. It was observed that the lncRNAs were the most reported ncRNAs associated with drug response of BCa. This review paves the way of introducing a prognostic panel of ncRNAs for the BCa patients to improve the selection of an efficient chemotherapeutic strategy based on ncRNA profile of BCa patients.

## Data Availability

The datasets used and/or analyzed during the current study are available from the corresponding author on reasonable request.

## Abbreviations

NMIBC: Non-Muscle-Invasive Bladder Cancer
BCa: Bladder cancer
ncRNAs: Non-coding RNAs
MIBC: Muscle-invasive bladder cancer
BCG: Bacille Calmette-Guérin
lncRNA: Long non-coding RNAs
miRNA: Micro RNAs
circRNA: Circular RNAs
SIRT1: Sirtuin-1
HMGA1: High-mobility group A1
IGF-1R: Insulin-like growth factor-1 receptor
UCA1: Urothelial carcinoma associated 1
EMT: Epithelial-mesenchymal transition
GHET1: Gastric carcinoma proliferation-enhancing transcript 1
Dox: Doxorubicin
NET1: neuroepithelial cell transforming 1
GSH: Glutathione
COX-2: Cyclooxygenase-2
GOLPH3: Golgi phosphoprotein 3
ABC: ATP-binding cassette
OS: Overall survival
PSEN1: Presenilin
C/EBP: CCAAT/enhancer binding protein
IGF: Insulin growth factor
